# Bone biomaterials and interactions with stem cells

**DOI:** 10.1038/boneres.2017.59

**Published:** 2017-12-21

**Authors:** Chengde Gao, Shuping Peng, Pei Feng, Cijun Shuai

**Affiliations:** 1 State Key Laboratory of High Performance Complex Manufacturing, College of Mechanical and Electrical Engineering, Central South University, Changsha, China; 2 The Key Laboratory of Carcinogenesis of the Chinese Ministry of Health, Xiangya Hospital, Central South University, Changsha, China; 3 The Key Laboratory of Carcinogenesis and Cancer Invasion of the Chinese Ministry of Education, Cancer Research Institute, Central South University, Changsha, China; 4 Jiangxi University of Science and Technology, Ganzhou, China; 5 Key Laboratory of Organ Injury, Aging and Regenerative Medicine of Hunan Province, Xiangya Hospital, Central South University, Changsha, China

## Abstract

Bone biomaterials play a vital role in bone repair by providing the necessary substrate for cell adhesion, proliferation, and differentiation and by modulating cell activity and function. In past decades, extensive efforts have been devoted to developing bone biomaterials with a focus on the following issues: (1) developing ideal biomaterials with a combination of suitable biological and mechanical properties; (2) constructing a cell microenvironment with pores ranging in size from nanoscale to submicro- and microscale; and (3) inducing the oriented differentiation of stem cells for artificial-to-biological transformation. Here we present a comprehensive review of the state of the art of bone biomaterials and their interactions with stem cells. Typical bone biomaterials that have been developed, including bioactive ceramics, biodegradable polymers, and biodegradable metals, are reviewed, with an emphasis on their characteristics and applications. The necessary porous structure of bone biomaterials for the cell microenvironment is discussed, along with the corresponding fabrication methods. Additionally, the promising seed stem cells for bone repair are summarized, and their interaction mechanisms with bone biomaterials are discussed in detail. Special attention has been paid to the signaling pathways involved in the focal adhesion and osteogenic differentiation of stem cells on bone biomaterials. Finally, achievements regarding bone biomaterials are summarized, and future research directions are proposed.

## Introduction

As an important tissue/organ in the human body, the bone plays a vital role in not only protecting the organs inside the body but also providing mechanical support, hematopoiesis, and mineral storage.^1–3^ Moreover, it can coordinate with muscular tissue to accomplish various movements and respond to environmental changes.^4^ Although bone has a certain capability for regeneration and self-repair,^5^ large segmental bone defects caused by severe trauma, tumor resection, cancer, or congenital diseases can only be repaired by bone grafting.^6^ In recent years, there has been an increasing demand for bone biomaterials, which are also called bone graft substitutes.^7^ In the United States, over 2 million surgeries are conducted each year to repair damaged or fractured bones by grafting. As a result, the bone biomaterial market in the United States exceeded 39 billion dollars in 2013.^8^ In China, the number of patients with limited limb function due to bone defects has reached up to 10 million.^9^ However, many patients cannot be treated effectively due to the lack of bone biomaterial availability. Consequently, they must settle for less desirable options, such as amputation due to bone tissue necrosis, which places a great burden on both the patients and society.^10^ Therefore, bone defects have become a serious social problem, and more effort should be devoted toward developing bone biomaterials for bone repair.^11^

**Figure 1:**
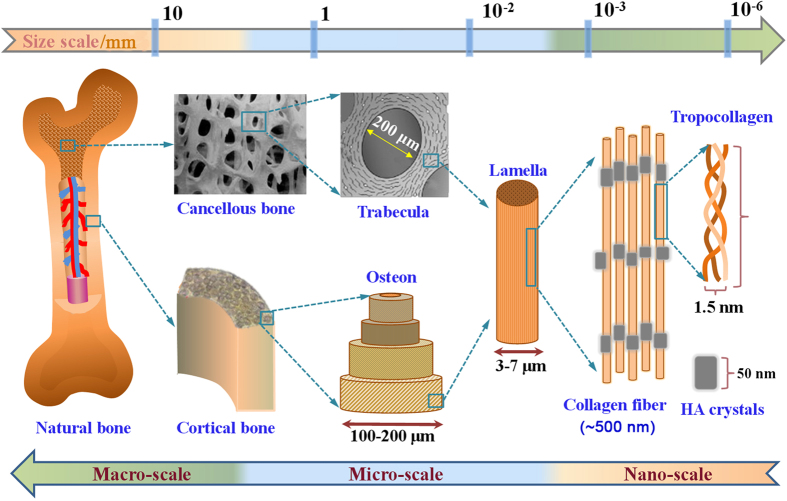
The chemical composition and multi-scale structure of natural bone.

The structure of natural bone is shown in Figure 1. In terms of composition, natural bone is a composite material composed of organic and inorganic materials.^12^ The organic materials are mainly collagen fibers containing tropocollagen, which endow the bone with a certain toughness.^13^ The inorganic materials are mainly calcium (Ca) and phosphorus (P) in the form of hydroxyapatite (HA) crystals, as well as sodium (Na), potassium (K), magnesium (Mg), fluoride (F), chlorine (Cl), carbonate (CO_3_^2−^), and some trace elements, such as silicon (Si), strontium (Sr), iron (Fe), zinc (Zn), and copper (Cu), which endow the bone with a certain strength.^14^ In terms of structure, natural bone has a multi-scale structure that can be divided into cortical bone and cancellous bone.^15^ Cortical bone is located at the surface of the bone and contains 99% of the Ca and 90% of the phosphate in the human body. It is relatively dense and strong, with a low porosity of 5%–10%.^16^ Cancellous bone is spongy, and this tissue is distributed inside the bone. It is formed by intertwining lamellar trabeculae, which contain hematopoietic cells, adipose tissue, and blood vessels. Cancellous bone accounts for only 20 wt% of the bone in the human body, but its porosity reaches 50%–90%, with a specific surface area almost 20 times that of cortical bone.^17^ These special compositions and structures endow bone with superior properties to accomplish various functions. However, the composition and structure of bone vary with the defect site, age, genetic inheritance, and living conditions of patients, resulting in different demands for bone implants.^18^ Therefore, it has long been a challenge to develop ideal bone biomaterials that meet the requirements for bone repair.

As a bridge between native tissues and seeded cells, bone biomaterials play a vital role in bone repair.^19^ The specific biomaterial and porous structure can guide and control the type, structure, and function of regenerated tissue.^20^ To obtain a composition, structure, and function similar to that of natural bone, the following issues regarding bone biomaterials should be addressed: (1) developing ideal biomaterials with appropriate biological properties and mechanical performance. Bone biomaterials should primarily meet safety requirements, such as being non-toxic and not eliciting inflammatory or immune responses. Moreover, they should possess good biocompatibility and bioactivity, as well as controllable biodegradability.^21^ Furthermore, bone biomaterials should not simply fill the bone defects but should also degrade continuously ***in vivo***. To avoid compromising the function and structure of new bone, the degradation rate of bone biomaterials should match the growth rate of new bone.^22,23^ Additionally, bone biomaterials should possess mechanical properties and stability appropriate for the defect site because biomaterials need to provide structural support for both the defect site and the newly formed tissue.^24,25^ (2) Constructing a cell microenvironment with pore sizes on the nano-, submicro-, and microscale. A multi-scale porous structure can provide the necessary space and environment for the growth of different cells and tissues and facilitate extracellular matrix (ECM) formation, nutrient transport, and nerve and blood vessel ingrowth.^26^ Since the site and structure of bone defects differ among patients, bone biomaterials should have a customizable external shape and internal structure to match the defect site and provide long-term stability.^27^ (3) Inducing the oriented differentiation of stem cells for artificial-to-biological transformation. Positive interactions between bone biomaterials and cells are necessary to facilitate cell functions, such as adhesion, proliferation, differentiation, and gene expression.^28^ More importantly, bone biomaterials should have good osteoinductivity, that is, the ability to induce the differentiation of stem cells into osteogenic cells, leading to the formation of bone tissue.^29^ Good osteoinductivity can provide a compatible interface between a bone biomaterial and native tissue and guide the growth of bone tissue along the interface, eventually resulting in the formation of new bone tissue closely integrated with the bone biomaterial.^30^ Therefore, the above three issues have been intensely investigated over recent years.

## Main types of bone biomaterials

After implantation, bone biomaterials act as a medium for the contact and interaction of bone implants with the surrounding cells/tissues. Thus, the selection of bone biomaterials is a key step in the preparation of ideal bone implants.^31^ Generally, the selection of bone biomaterials is based on their inherent biocompatibility, biodegradability, and mechanical properties, as well as the resulting cell behavior. In addition, the physicochemical characteristics, molecular weight, and hydrophilicity/hydrophobicity of bone biomaterials are also very important.^32^ As mentioned above, natural bone is composed of cortical and cancellous bone, which exhibit quite different mechanical properties because of their different compositions and structures. It has been reported that the compressive strength, fracture toughness, and elastic modulus of cortical bone are within 100–180 MPa, 2–12 MPa·m^1/2^, and 7–30 GPa, respectively, while those of cancellous bone are within 2–12 MPa, 0.1–0.8 MPa·m^1/2^, and 0.2–0.5 GPa, respectively.^33–35^ To provide sufficient mechanical support for the defect site and newly formed tissues during bone repair, bone implants should possess mechanical properties that match the natural bone; otherwise, bone repair failure may occur in the human body.^36,37^ Therefore, the mechanical properties of bone biomaterials are considered one of the most important selection criteria. Commonly studied bone biomaterials can be categorized as bioceramics, polymers, and biomedical metals.^38^ It has been widely accepted that bioceramics are brittle materials with a low fracture toughness, insufficient mechanical strength, and a high elastic modulus compared with those of cortical bone, while the mechanical strength and elastic modulus of polymers are far below the requirements of cortical bone,^39,40^ which significantly limits their application in weight-bearing sites. In the past decade, intensive efforts have been devoted to improving the mechanical properties of bioceramics or polymers by creating composites with other bone biomaterials, but limited improvements have been achieved due to the difficulties in obtaining a uniform dispersion and strong interfacial bonding.^41–44^ As a result, currently, bioceramics and/or polymers can only be used as bone stuffing or for the reconstruction of bone defects in cancellous bone, instead of direct weight-bearing applications.^45^ In contrast, biomedical metals generally have a higher mechanical strength than bioceramics and polymers, but their mechanical properties, especially the elastic modulus, are also incompatible with that of natural bone, causing stress shielding and further bone loss, bone relaxation, and osteoporosis.^46,47^ Therefore, the mechanical properties of current bone biomaterials can not meet the requirements for bone repair, especially in weight-bearing applications.

Two other important criteria for bone biomaterials are biodegradability and bioactivity. Some bioceramics (including aluminum oxide and zirconium oxide), polymers (including polyurethane and silicone rubber), and biomedical metals (including stainless steel and titanium (Ti) alloys) are non-biodegradable and bioinert materials. Despite good biocompatibility and/or excellent mechanical strength, they cannot biodegrade and thus are retained as permanent implants **in vivo**.^48^ Moreover, these biomaterials lack bioactivity and can only bond with bone tissue through mechanical interlocking, which easily results in loosening and wear after long-term implantation.^49–51^ Therefore, recent studies have gradually shifted their focus onto biodegradable and/or bioactive materials, including bioactive ceramics, biodegradable polymers, and biodegradable metals.^52–55^

### Bioactive ceramics

#### Types and characteristics

Bioactivity, first proposed by Professor Hench in 1969, is a characteristic of chemical bonding between bone biomaterials and biological tissues.^56^ To date, bioactive materials mainly refer to bioactive ceramics, such as Ca–P ceramics, Ca–Si ceramics, bioactive glasses, and calcium sulfates.^52,57^

As the most representative bioactive ceramics, Ca–P ceramics, including HA [Ca_10_(PO_4_)_6_(OH)_2_] and tricalcium phosphate [TCP, Ca_3_(PO_4_)_2_], have compositions similar to that of natural bone.^58^ They not only have good biocompatibility and osteoconductivity but can also osseointegrate with the defect site.^59^ Moreover, the degradation products and released ions can participate in the human metabolism and create an alkaline environment to enhance cell activity and accelerate bone repair.^60^ HA is the main mineral of natural bone and thus has been extensively studied as a bone biomaterial.^61–63^ It has a theoretical density of 3.16 g·cm^−3^ and a Ca/P molar ratio of 1.67.^63^ After implantation, it is capable of guiding new bone growth and forming chemical bonding with bone tissue with a bonding strength 5–7 times higher than that between bioinert ceramics and natural bone.^64^ Meanwhile, HA has good osteoinductivity, and its hydroxyl groups have an ideal affinity for the amino acids, proteins, and organic acids in the human body via hydrogen bonding.^65^ However, HA has a relatively high crystallinity and stability, which makes it difficult to degrade **in vivo**.^66^ Compared with HA, TCP is more degradable and becomes soluble more rapidly; its degradation rate is 10–20 times higher than that of HA.^66^ The degradation products can provide abundant Ca and P for osteoblasts and induce bone regeneration.^67^ Furthermore, TCP can form a direct connection with the bone tissue after implantation, without the intervention of fibrous connective tissue.^68^ Bruder *et al*^69^ implanted porous TCP seeded with bone marrow osteoblasts into dogs. After 3 months, the implants had integrated with the host bone, and the bone defects were repaired.

In recent years, Ca–Si ceramics, including wollastonite (CaSiO_3_), akermanite (Ca_2_MgSiO_7_), diopside (CaMgSi_2_O_6_), hardystonite (Ca_2_ZnSi_2_O_7_), bredigite (Ca_7_MgSi_4_O_16_), and merwinite (Ca_3_MgSi_2_O_8_), have been developed.^70^ Ca exists in the active area of natural bone and plays an important role in the growth of bones and blood vessels.^71^ Si is one of the most important trace elements in the human body, and its content reaches 100 ppm in bones and 200–550 ppm in the ECM.^72^ Si is commonly absorbed in the form of metasilicate, which is widely distributed in connective tissue.^73^ Si plays an important role in bone calcification and is beneficial for improving bone density and preventing osteoporosis.^72^ Especially in the early stage of osteogenesis, a high Si content in new bone can increase the degree of calcification to some extent, while a Si deficiency always causes bone distortion.^74^ Some studies have shown that Si can not only promote the synthesis of collagen and proteoglycan but also activate bone-related gene expression and stimulate osteoblast proliferation and differentiation.^75^ It has been reported that the ion products released by Ca–Si ceramics can enhance the effectiveness of insulin-like growth factor (IGF) II, which is specifically related to cell proliferation.^76^ This enhancement is achieved by inducing the transcription of growth factors and carrier proteins and regulating the separation of binding proteins. Wu *et al*^77^ prepared porous akermanite by a polymer foaming method and analyzed the effect of ion release on cell activity. The results showed that Ca and Si ions could be released from akermanite during the immersion process, which promoted the early mineralization, division, and gene expression of osteoblasts. Moreover, Si ions showed better stimulatory effects on cells than Ca ions. Compared with Ca–P ceramics, Ca–Si ceramics have shown better performance in terms of mechanical properties.^78–81^ For example, most Ca–Si ceramics, such as diopside and akermanite, have shown a higher bending strength and fracture toughness than HA.^82^ Moreover, the relatively wider range of Ca–Si ceramics than Ca–P ceramics in chemical compositions also has a significant impact on regulating the mechanical properties of bioactive ceramics.^83^ Mechanical stability is also an important characteristic of bone implants because they should maintain sufficient mechanical strength to provide support for new tissues before completely degrading.^84^ It was reported that diopside had a better compressive strength and mechanical stability than bioactive glasses and wollastonite.^85^ More specifically, the initial compressive strengths (0.63–1.36 MPa) of diopside scaffolds with porosities of 75%–80% were comparable to that of cancellous bone and lower than that of cortical bone. After 14 days of degradation in simulated body fluid (SBF), the compressive strengths of diopside scaffolds were reduced by 30%, which was much less than the reduction observed for bioactive glass scaffolds (54%) and wollastonite scaffolds (60%) under the same conditions. These features demonstrate the great potential of Ca–Si ceramics for application in bone repair.

Bioactive glasses mainly consist of silicon dioxide (SiO_2_), phosphorus pentoxide (P_2_O_5_), and calcium oxide (CaO), and some of them also contain sodium oxide (Na_2_O), potassium oxide (K_2_O), and/or magnesium oxide (MgO).^86^ Based on the differences in composition and proportion, bioactive glasses can be categorized as 45S5 (molar ratio: 46.1% SiO_2_, 24.4% Na_2_O, 26.9% CaO, and 2.6% P_2_O_5_), 58S (molar ratio: 60% SiO_2_, 36% CaO, and 4% P_2_O_5_), and 1393 (molar ratio: 54.6% SiO_2_, 22.1% CaO, 6.0% Na_2_O, 1.7% P_2_O_5_, 7.9% K_2_O, and 7.7% MgO), among others.^87^ Bioactive glasses have good biocompatibility and an excellent capability for heterogeneous nucleation and apatite deposition. They can form strong chemical bonding with surrounding bone tissue within a short time.^88^ In the physiological environment, bioactive glasses release Si, Ca, P, and Na ions through ion exchange, leading to the formation of electronegative Si-OH and a Si-rich gel layer on the surface through polycondensation.^89^ On the one hand, the electronegative Si-OH bonds with different types of proteins via hydrogen and ammonia bonding, resulting in high-density protein adsorption. On the other hand, Ca^2+^, PO_4_^3−^, CO_3_^2−^, and OH^−^ in body fluids can be absorbed to form a hydroxycarbonate-apatite (HCA) layer on the surface of the Si-rich gel layer.^89^ The Si-rich gel layer and HCA layer each possess a high surface areas and are thus capable of adsorbing large quantities of biomolecules, thereby promoting extracellular responses.^89^ More importantly, as bioactive glasses are by far the only bioceramics that can form bonding with both hard and soft tissues,^90^ they have received great attention as bone biomaterials. Sepulveda *et al*^91^ found that differences in the type and powder properties of bioactive glasses can generate different degradation rates and bioactivities, and thus modulate the release of active ions. Compared with 45S5, 58S had a faster degradation rate and can be completely covered by a high-crystallinity apatite layer after only 1 h of immersion. The degradation rates of both bioactive glasses increased with decreasing powder particle size.

#### Disadvantages and strategies

Bioactive ceramics have good biocompatibility and bioactivity and have thus become an important focus in bone biomaterial research. However, there are still some shortcomings in the use of bioactive ceramics as bone biomaterials, such as low toughness and insufficient strength. In particular, the strength of bioactive ceramics is further weakened when used to form porous implants.^92^ Therefore, currently, bioactive ceramics can only be used in non- or low-loading applications. To address these problems, researchers have proposed the use of nanoscale second phases,^93^ surface coatings,^94^ and self-toughening methods^95^ to improve the mechanical properties of bioactive ceramics.

**Figure 2:**
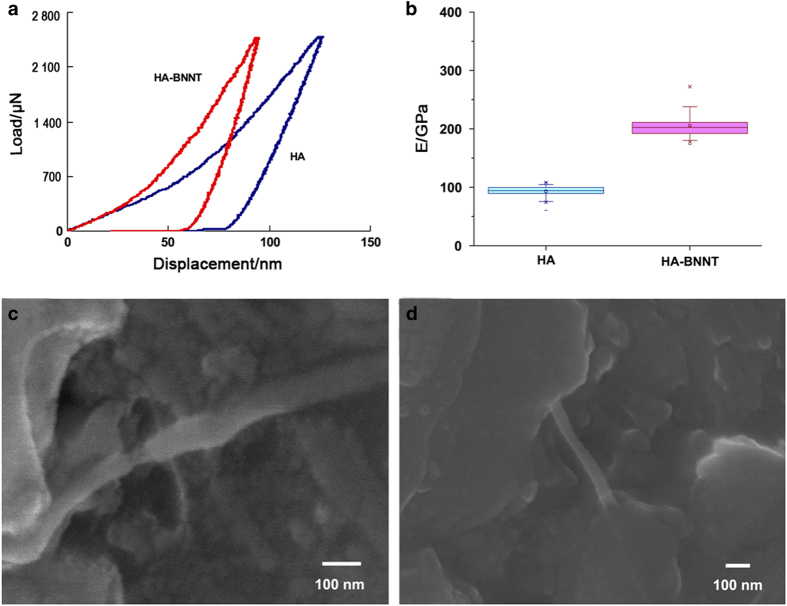
(**a**) Load vs displacement plot and (**b**) elastic modulus for HA and BNNT/HA obtained by nanoindentation. (**c**) Sword-in-sheath phenomenon and (**d**) bridging mechanism of BNNTs.^111^

The commonly used nanoscale second phases include nanoparticles, nanotubes, and nanosheets. In terms of the dimension of reinforcement, nanoparticles are zero-dimensional reinforcements and are the most popular method for reinforcing ceramics.^96^ Nanoparticles possess a large specific surface area and chemical activity^97^ and can thus reinforce materials effectively. The typical reinforcement mechanisms of nanoparticles can be summarized as follows: (1) the pinning effect:^98^ when encountering nanoparticles, the crack tip cannot pass through and is deflected because of the pinning effect, which dissipates the fracture energy and thus enhances the mechanical properties of bioactive ceramics. (2) Dispersion reinforcing:^99^ the dispersed nanophase can generate local stress to enhance bioactive ceramics through the mismatch of physicochemical properties, such as Young’s modulus and thermal expansion coefficient. (3) Grain refinement:^100^ nanoparticles can inhibit the abnormal growth of ceramic grains, resulting in a uniform and refined grain structure. (4) Fracture mode transformation:^101^ the nanoparticles act as a nucleus and are wrapped by ceramic particles to form an intragranular structure, which weakens the main grain boundary and contributes to transgranular fracture when the bone biomaterial fractures. In comparison, nanotubes and nanosheets are one- and two-dimensional reinforcements, respectively.^102^ In recent years, they have attracted increasing attention in ceramic-based composites owing to their large length/diameter and width/thickness ratios, respectively.^103^ Commonly used nanotubes and nanosheets include carbon nanotubes (CNTs) and graphene.^104–107^ Their reinforcement mechanisms mainly include the following: (1) crack deflection:^108^ when encountering nanotubes or nanosheets, the crack cannot pass through and can only deflect to continue propagating, which prolongs the path and increases energy consumption for crack propagation. (2) Crack-bridging:^109^ nanotubes or nanosheets bridge both sides of the crack to prevent further propagation and opening. (3) The pull-out effect:^110^ nanotubes or nanosheets are pulled out from the ceramic matrix during crack propagation, which consumes external energy by interfacial friction. Lahiri *et al*^111^ added boron nitride nanotubes (BNNTs) to HA ceramics and found that the mechanical properties were improved by the sword-in-sheath phenomenon and crack-bridging mechanism of the BNNTs (Figure 2). In addition, many studies have confirmed that the large specific surface area and unique structure of nanoscale second phases can not only improve mechanical properties but also promote cell adhesion and proliferation on bioactive ceramics.^112,113^

Another reinforcement method is surface treatments to form coatings on the surface of bioactive ceramics. Surface coatings can passivate stress concentration and inhibit crack propagation in ceramics, leading to transformation of the fracture mode from integral fracture to layered fracture and thus enhancing the mechanical properties.^114^ Commonly used coating materials include polymers^115^ and glasses.^116^ Milovac *et al*^115^ coated polycaprolactone (PCL) on porous HA by vacuum impregnation and found that the compressive strength and elastic modulus of the coated HA were close to that of trabecular bone. Roohani-Esfahani *et al*^116^ coated 58S on the surface of HA/TCP biphasic ceramics and found that the compressive strength and elastic modulus were increased by 14 and 3 times, respectively. Self-toughening is another reinforcement technology via microstructure design.^117^ Specifically, raw materials that can generate a second phase are introduced to form whiskers or grain reinforcements with a high length/diameter ratio by *in situ* growth on ceramic grains. Zhou *et al*^118^ prepared HA whiskers on HA substrates by the *in situ* growth method. The results showed that the compressive strength of HA was greatly improved and that the fracture mode changed from the original integral, brittle fracture mode to a two-step fracture mode.

### Biodegradable polymers

#### Types and characteristics

Biodegradable polymers degrade mainly through microbial and enzymatic actions and the mechanical damage caused by cell growth.^119^ They can be categorized as natural biodegradable polymers and synthetic biodegradable polymers.^120,121^

Natural biodegradable polymers are directly derived from animal or plant tissues and mainly include collagen, chitosan, and cellulose.^122,123^ The main advantages of these biomaterials lie in their low risk of eliciting an immune response, non-toxicity, few side effects, and wide availability, as well as their good bioactivity, cell affinity, and hydrophilicity.^124^ Their degradation products are mostly amino acids, which can be absorbed directly by the human body.^125^ As one of the most important extracellular components, collagen constitutes the framework of the ECM, provides necessary elasticity for cells, and plays an important role in cell migration and growth.^126^ O'Loughlin *et al*^127^ seeded cells on collagen implants prepared by a freeze-drying method, and the **in vivo** results showed that collagen implants facilitated cell migration and blood vessel formation. Su *et al*^128^ loaded bone morphogenetic protein 2 (BMP-2) and dexamethasone (Dex) in collagen composite fibers and found that both growth factors could be stably released along with the degradation of the material, which promoted the differentiation of bone marrow mesenchymal stem cells (BMSCs) into osteoblasts. Chitosan, generally derived from chitin, is the only type of alkaline aminopolysaccharide.^129^ The structure and properties of chitosan are very similar to those of the aminopolysaccharides in the ECM. Its surface is rich in positive charges, which is favorable for the adhesion of negatively charged cells.^130^ Moreover, the surface of chitosan contains many pendant groups, which can be modified (such as via sulfonation, esterification, and etherification) for specific needs.^131^ Seol *et al*^132^ prepared porous chitosan by a freeze-drying method and performed cell culture experiments using rat calvarial osteoblasts. The results showed that chitosan was beneficial for the proliferation and differentiation of osteoblasts. Moreover, calcium deposition and new bone formation were observed after cells were cultured on chitosan. Similar findings were also reported by Klokkevold *et al*,^133^ who demonstrated that chitosan could promote the differentiation of osteoblasts.

Synthetic biodegradable polymers include poly(lactic acid) (PLA),^134^ poly(glycolic acid) (PGA),^135^ poly(lactic-co-glycolic acid) (PLGA),^136^ poly(vinyl alcohol) (PVA),^137^ PCL,^138^ and polyoxyethylene (POE). They greatly differ from each other in terms of structure and properties.^139^ Thus, the surface properties and degradation behavior can be modulated via the molecular design and synthesis process, resulting in synthetic biodegradable polymers being very versatile as bone biomaterials.^140^ Compared with natural biodegradable polymers, numerous varieties of synthetic biodegradable polymers are available, and they can provide improved mechanical properties and plasticity.^141^ In the physiological environment, the surface of polymers undergoes a hydrolysis reaction; as a result, the polymer chains are cut into compounds with smaller molecular weights. These compounds are either absorbed or excreted by the body.^142^ PLA is one of the most typical synthetic biodegradable polymers, and it is derived from renewable plant resources.^143^ It has good biocompatibility and biodegradability, and the degradation products are not toxic to the human body.^144^ PLA also possesses a tensile strength up to 40–60 MPa and an elastic modulus of 3 000–4 000 MPa. Therefore, it has been used to replace stainless steel as a new type of orthopedic fixation material in Europe and the United States.^145^ PLA has three stereoisomers: poly(l-lactic acid) (PLLA), poly(d-lactic acid) (PDLA), and poly(d,l-lactic acid) (PDLLA).^146^ They have good plasticity and processability and can be prepared into 3D implants by methods such as electrospinning,^147^ gas foaming,^148^ and solution casting.^149^ Therefore, PLA has been widely used in bone repair, vascular substitutions, and other biomedical applications. Suryanegara *et al*^150^ evaluated the stability, biocompatibility, and degradability of PLA and found that PLA had good thermal stability and cytocompatibility. PGA is a semi-crystalline synthetic polymer with good biocompatibility and biodegradability.^151^ When it degrades, the non-crystalline part first degrades into glycolic acid (GA), which can be easily metabolized by the body; then, the crystalline part degrades into harmless water and carbon dioxide.^152^ PLGA is randomly polymerized by two kinds of monomers, lactic acid (LA) and GA. It possesses good biocompatibility and modular biodegradability because the degradation rate increases with the GA/LA ratio in PLGA.^153,154^ This modular degradation behavior has enabled it to be widely used in the biomedical field. Currently, PLA, PGA, and PLGA are approved by the US Food and Drug Administration for clinical applications, including surgical sutures, injection capsules, and bone biomaterials.^155^ PVA can degrade **in vivo**, and the degradation products are not toxic to the human body.^156^ PVA also has good hydrophilicity and excellent chemical stability, and its semi-crystalline characteristics can provide the necessary channels for oxygen and nutrient transport, as well as metabolic waste excretion.^157^ PCL is a semi-crystalline polymer formed by the ring-opening polymerization of caprolactone. It has excellent thermal processability and a slow degradation rate.^158^ Compared with most biodegradable polymers, PCL has better mechanical properties and higher break elongation.^159^ Williams *et al*^160^ prepared a porous PCL to mimic the jawbone, and the **in vitro** osteogenic results showed that after 1 month, normal bone structures, including osteoblasts, bone protrusion, and bone marrow space, were observed in both the inner and outer part of the porous PCL. POE is synthesized by the polycondensation of polybasic acid or polybasic ester with polyol under anhydrous conditions.^161^ It is a hydrophobic polymer and degrades by ester bond hydrolysis due to the infiltration of water molecules into POE, followed by surface and bulk degradation.^161^ The resulting water-soluble small molecules can be metabolized by the host organism.

#### Disadvantages and strategies

Although biodegradable polymers have been widely used, several problems remain: (1) there is a contradiction between the mechanical properties and degradation rate, that is, a high molecular weight usually accompanies a high strength, while the degradation rate can hardly meet the requirements for bone repair.^138^ (2) Natural polymers have poor thermal stability and processability and poor degradation rate control. (3) Synthetic polymers exhibit poor cell adhesion, which is mainly attributed to the fact that polymers have few polar groups and thus an extremely low surface free energy.^162^ (4) Synthetic polymers lack bioactivity and cannot form chemical bonding with human tissue.^163^ (5) The degradation products of synthetic polymers are generally mildly acidic, and local acidity that is too high will hinder the cell growth on bone biomaterials or even cause inflammation;^164^ for example, clinical studies indicate that the ratio of nonspecific inflammation caused by PLA and PGA is up to 8%.^165^ (6) There is still a gap in terms of the mechanical properties of biodegradable polymers and cortical bone, especially in hardness and strength.^166^ To address these problems, researchers have conducted intensive studies with a focus on creating biomaterials with excellent bioactivity and/or mechanical properties.

One research direction similar to that of bioactive ceramics is nanoscale second-phase reinforcing.^167,168^ Zhang *et al*^40^ incorporated surface-modified nanodiamond into PLLA and found that 10 wt% nanodiamond significantly improved the fracture strain and fracture energy of PLLA by 280% and 310%, respectively, compared with pure PLLA. Moreover, the **in vitro** mineralization results revealed that the apatite-forming ability of the composites was significantly improved. Nanodiamond was also introduced into PVA to improve the hardness and elastic modulus.^169^ Wang *et al*^170^ studied the effect of CNTs on the mechanical properties of PCL and found that the elastic modulus and tensile strength of PCL were enhanced by the pull-out effect of the CNTs. Khan *et al*^171^ reinforced PVA with boron nitride nanosheets (BNNSs) and found that a strong interface formed between them, resulting in a 40% increase in the modulus and strength of PVA with 0.12 vol% BNNSs.

**Figure 3:**
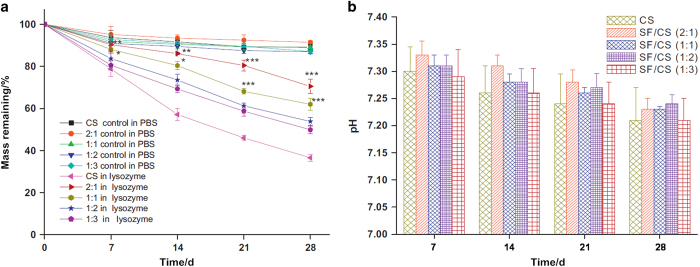
(**a**) Degradation behavior of silk fibroin protein/chitosan in 0.05 M phosphate-buffered saline (PBS) solution containing 1.6 μg·mL^−1^ lysozyme and in pure PBS solution (pH 7.4). (**b**) pH changes of the resultant solution. ***, **, and * indicate significant differences between groups at *P*<0.001, *P*<0.01 and *P*<0.05, respectively. The results showed that the biodegradation and stability of chitosan could be modified by silk fibroin protein.^181^

Another method to improve the properties of biodegradable polymers is the formation of composites with bioactive ceramics and/or other polymers.^172–174^ Duan *et al*^175^ prepared composite bone biomaterials by a combination of biodegradable poly(3-hydroxybutyrate-co-3-hydroxyvalerate) and bioactive TCP, which provided a cell microenvironment for the adhesion, proliferation, and differentiation of osteoblasts. Nie *et al*^176^ incorporated HA into a PLGA matrix to prepare HA/PLGA composites and loaded them with BMP-2. The results showed improved cell adhesion on the HA/PLGA composites and that the composites could continuously release BMP-2. Cui *et al*^177^ prepared PDLLA/HA composites through the *in situ* growth of HA in PDLLA, and a stable interfacial bonding was formed between them, leading to an improved tensile strength and Young's modulus. In addition, the composites also exhibited favorable apatite formation and could maintain an active region at the interface for significantly improved cell differentiation. Bioactive glasses have also been incorporated into PCL, resulting in improved mechanical properties and apatite formation.^178,179^ Additionally, some studies have demonstrated that different degradation rates could be obtained by adjusting the ratios of the biomaterials in bone implants.^180^ For example, Bhardwaj *et al*^181^ prepared porous polymer composites with silk fibroin protein and chitosan, and degradation tests showed a decreased chitosan degradation rate after the addition of silk fibroin protein (Figure 3), which was attributed to an enhanced steric hindrance effect via physical interactions between them. Regarding the acidic degradation products, an attempt has been made to neutralize the acidic products by adding alkaline materials to polymers, and good results have been achieved.^182,183^ Saravanan *et al*^184^ fabricated chitosan/nano-HA/nano-silver composites based on the following considerations: first, chitosan has good biodegradability and biocompatibility, as well as the potential to be modified by various chemical modifications to obtain desired properties; second, nanoscale HA can improve protein absorption and cell adhesion on bone biomaterials and can provide significant mechanical reinforcement; third, metallic nanoparticles, such as Cu, Zn, and silver (Ag), can prevent infections via their antibacterial activity. The results indicated that ternary composites had better mechanical strengths and degradation rates than binary composites. Moreover, they were non-toxic to osteoblasts and exerted broad-spectrum antibacterial activity against Gram-negative and Gram-positive bacteria.

### Biodegradable metals

#### Types and characteristics

Biodegradable metals, mainly including Mg, Zn, Fe, and their alloys, are considered potential load-bearing bone biomaterials due to their better toughness and processability than bioactive ceramics and better strength and stiffness than biodegradable polymers.^185,186^ Among these biodegradable metals, Mg and its alloys have attracted the most attention because their Young's moduli (~45 GPa) and densities (~1.74 g·cm^−3^) are close to those of cortical bone (Young's modulus ~3–25 GPa and density ~1.8–2.0 g·cm^−^^3^).^186–191^ Thus, they can effectively relieve the stress-shielding effect. Moreover, Mg is a major element in the human body and can activate a variety of enzymes involved in metabolic processes.^47,192^ The daily demand for Mg is 300 mg in adults and 250 mg in children.^189^ As bone biomaterials, they can not only provide mechanical support at the initial stage of implantation but also degrade along with the growth of new bone tissue. Mg can degrade into Mg ions **in vivo** and then be absorbed by the surrounding tissues or excreted through the metabolism.^193^ Thus, Mg and its alloys have been widely considered revolutionary metallic biomaterials.^194^

#### Disadvantages and strategies

As a bone biomaterial, the degradation rate of Mg and its alloys should be <0.5 mm per year to provide an effective service period of at least 12 weeks.^47^ However, Mg has active chemical properties with a low standard corrosion potential of −2.37 V,^195^ and the surface oxide film formed in corrosive medium exhibits a porous structure (Pilling–Bedworth ratio=0.8).^188^ These two factors result in the rapid degradation of Mg and its alloys, especially in an environment containing high Cl ion concentration.^196^ On the one hand, degradation that is too rapid leads to the quick loss of implant mechanical integrity and stability.^197^ On the other hand, fast degradation leads to the rapid release of large amounts of hydrogen, which aggregates around the implant to form bubbles.^198^ These bubbles inevitably impair the physiological function of the surrounding tissue and the regeneration of the defect site.^198^ Moreover, rapid degradation also causes an increase in the alkalinity of body fluids, leading to hemolysis or even osteolysis.^199^

In general, the degradation of Mg and its alloys in the physiological environment occurs via a corrosion process. The main corrosion mechanisms can be summarized as intergranular corrosion, galvanic corrosion, and pitting corrosion. Intergranular corrosion occurs due to the different chemical compositions of the grain boundaries and interior.^200^ This significantly weakens the interfacial bonding between Mg grains, thus deteriorating the mechanical strength.^201,202^ Lin *et al*^203^ revealed that intergranular corrosion and pitting corrosion coexisted on the surface of an extruded ZAX1330 Mg alloy [12.83 wt% Zn, 3.35 wt% aluminum (Al), 0.20 wt% Ca] when immersed in SBF for 24 h. After heat treatments, the intergranular corrosion was relieved due to changes in the grain size and second phase distribution. The galvanic effect easily occurs due to the corrosion potential difference between the Mg matrix and second phases or impurities.^204^ In this case, the Mg matrix acts as the anode and undergoes rapid galvanic corrosion.^205^ For example, a study by Coy *et al*^206^ showed that severe galvanic corrosion attacked an as-cast ZE41 Mg alloy due to the combined effects of cathodic precipitates Zr_4_Zn and Mg_7_Zn_3_REE [-REE refers to rare earth elements, such as lanthanum (La), gadolinium (Gd), and cerium (Ce)]. Li *et al*^207^ studied the dynamic corrosion process of as-cast AZ63 alloys, revealing that the corrosion initiates easily due to the formation of galvanic couples between the Mg matrix and the β-Mg_17_Al_12_ phase. Meanwhile, impurities, such as Fe, Cu, nickel, and cobalt, in the matrix may locally accelerate the hydrogen evolution reaction. Pitting corrosion is a typical localized corrosion mode.^208^ Pitting attack begins from an initial breakdown of certain points on the surface film, and then the corrosion pit develops laterally into a deep porous pit due to the continuous collapse of the pit fronts. Pitting corrosion resistance is believed to be closely related to the passivity of the surface film.^209^ Previous studies have revealed that a higher breakdown potential (*E*_b_) always leads to a high resistance to the passive film breakdown caused by pitting attack.^210,211^

**Figure 4:**
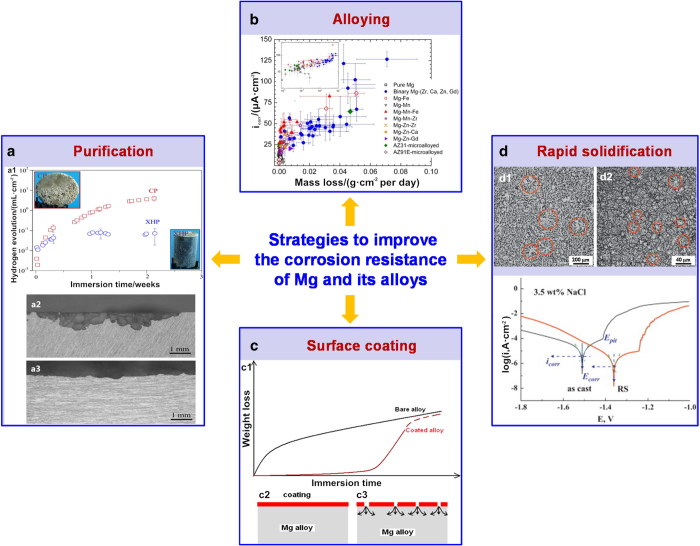
Commonly used strategies for improving the corrosion resistance of Mg and its alloys: (**a**) purification, (**b**) alloying, (**c**) surface coating, and (**d**) rapid solidification.^212–215^

To date, researchers have devoted extensive efforts to improving the degradation behaviors of Mg and its alloys. The strategies used can be summarized as purification, alloying, surface coating, and rapid solidification (RS) (Figure 4). Purification can improve the degradation behavior by reducing the galvanic corrosion between the Mg matrix and second phases or impurities.^216^ For example, highly pure Mg (0.004 5 wt% Fe, <0.002 wt% Cu, and <0.002 wt% Ni) has a significantly decreased corrosion rate compared with commercially pure Mg (0.02 wt% Fe, <0.002 wt% Cu, and <0.002 wt% Ni) in Hank’s solution.^217^ Purification could be realized by permanent mold direct chill casting,^218^ vacuum distillation,^219^ zone solidification,^220^ and sputter deposition.^221^ It should also be noted that the presence of elemental impurities in Mg is unavoidable. On the one hand, purification becomes less efficient as the purity level of Mg increases.^219^ On the other hand, highly pure Mg exhibits a relatively low mechanical strength for bone repair due to the low solid solubility of common metallic elements in the Mg matrix. For example, the yield tensile strength of casted commercially pure Mg is less than 50 MPa, which is lower than that of cortical bone.^222^

Alloying a small amount of an element, including Ca, Zn, Al, manganese (Mn), zirconium (Zr), Sr, and REEs, can alter the chemical composition of Mg and its alloys, as well as the distribution, volume fraction, and size of the second phase, which has significant effects on the degradation behavior.^223^ The main mechanisms of alloying for enhanced corrosion resistance can be described as follows: (1) the dissolved alloying elements favor an increase in the passivity of the surface film by forming a more sustainable, repairable, and protective film composed of the oxide or hydroxide of the alloying element, thus greatly improving the pitting corrosion resistance.^209,224–228^ (2) Some alloying elements contribute to reducing the volume fraction and size of the second phase or forming a new second phase with a potential closer to that of Mg, thus diminishing the galvanic corrosion effect.^229–231^ (3) The alloying elements may give rise to a continuously distributed second phase, which completely covers Mg grains and acts as a passive barrier to hinder the propagation of corrosion.^232,233^ (4) Some alloying elements could reduce the amount of impurities, such as Fe and Ni, thus mitigating the galvanic corrosion effect;^234^ for example, Zr can effectively dislodge Fe from the melt alloy by the precipitation of Fe_2_Zr or FeZr_2_.^216^ Mn, Al, and Si can also lower the Fe content in Mg and its alloys.^209^ (5) Some alloying elements can reduce the exchange current density of the cathodic or anodic reaction in Mg and its alloys.^235–237^

Surface coating is another method for improving the corrosion resistance by isolating Mg and its alloys from body fluids.^238^ The commonly used coating materials include polymers,^239,240^ bioceramics,^241–243^ and their composites.^244,245^ Razavi *et al* prepared a nanostructured akermanite coating^246^ and diopside coating^247^ by electrophoretic deposition. The results showed that the akermanite and diopside coatings not only enhanced the corrosion resistance of Mg alloys but also improved their **in vitro** bioactivity. Hiromoto *et al*^248^ fabricated HA and octacalcium phosphate coatings on an AZ31 alloy, and the results showed that a self-healing layer could be formed on the alloy surface by the deposition of Mg and Ca compounds, which effectively prevented further corrosion. Gao *et al*^249^ prepared a rod-like nano-HA and Mg_3_(PO_4_)_2_ composite coating on the surface of Mg alloys to seal the surface pores caused by micro-arc oxidation treatment, which improved the corrosion resistance. Although surface coatings can improve the corrosion resistance of Mg and its alloys to a certain extent, the uniformity and consistency of the coatings on the metal matrix still need to be improved.^238^ More importantly, the coatings can only protect Mg and its alloys in the early stage of implantation. After the removal of the coatings, the matrix will lose this protection and undergo rapid degradation.

RS technology has emerged as a promising method for improving the degradation behavior of Mg and its alloys.^215,250–252^ First, RS can effectively extend the solid solubility limits of alloying elements, thereby reducing the elemental impurities or harmful second phases.^253^ Second, RS generally results in a homogeneous microstructure, which could diminish the localized attack caused by the accumulation of cathodic phases at grain boundaries.^188^ Third, RS could contribute to refined grains and increased grain boundary density, which is favorable for increasing the surface passivation of Mg and its alloys.^254^ Aghion *et al*^254^ reported that the average corrosion rate (0.4 mm per year) of rapidly solidified AZ80 was slower than that of conventionally casted AZ80 (2.0 mm/year). The enhanced corrosion resistance was attributed to the increased Al content and consequent elimination of the β-phase in the matrix, which reduced the galvanic corrosion activity. A study by Hakimi *et al*^255^ revealed that the corrosion resistance of an Mg-6%Nd-2%Y-0.5%Zr alloy was significantly improved by RS due to the increased Nd_2_O_3_ in the external oxide layer, as well as a more homogeneous structure and reduced grain size. Despite the great potential of RS in improving the corrosion resistance of Mg and its alloys, relevant research is still in the initial stages of exploration. There are still many problems to be solved, such as element burning and dust evaporation during the RS process.

## Porous structure for the ECM microenvironment

Ideal bone biomaterials do not simply mimic the external shape and composition but should also match the internal structure of natural bone.^256^ In terms of structure, natural bone has a 3D architecture with a multi-scale porous structure ranging from the nanoscale to the submicro- and microscale,^257^ which offers a microenvironment for cell and tissue growth. The multi-scale porous structure not only provides a large number of binding sites for cell membrane receptors but also determines and maintains cell functionality.^258^ Cells can exhibit significantly different differentiation characteristics by sensing structural information.^259^ Specifically, a pore diameter of hundreds of micrometers (150–800 μm) can provide channels for the transport of nutrients and metabolites and is conducive to the ingrowth of new bone tissue and blood vessels.^260^ A pore diameter of tens of micrometers (10–100 μm) allows for the ingrowth of capillaries, thereby facilitating the exchange of nutrients and the discharge of metabolites.^261^ A nanoscale pore diameter can provide greater specific surface areas and more active targets, which are beneficial for cell adhesion and protein adsorption, thereby contributing to a favorable cell response.^260^ Therefore, in order to mimic the ECM microenvironment, bone biomaterials should have a multi-scale porous structure, and the pore diameter, shape, interconnectivity, and porosity not only determine the interactions between bone biomaterials and cells/tissue but also have important effects on the mechanical properties and degradation behavior.^262^ Generally, bone biomaterials should have an appropriate porosity and good pore interconnectivity because bone biomaterials without interconnected pores cannot provide the necessary channels for blood vessel growth.^263,264^ With further study on the interactions between bone biomaterials and organisms, people are expanding their requirements for bone biomaterials from biological and mechanical properties, such as biocompatibility, biodegradability, bioactivity, strength, and toughness, to the design and preparation of the internal porous structure and the modification of the material surface, thereby creating the specific physicochemical functions of bone biomaterials.^265–267^

### Pore diameters of hundreds of micrometers

To date, studies on the porous structure of bone biomaterials have mainly focused on a pore diameter of hundreds of micrometers. It has been reported that an open porous structure with a pore diameter of hundreds of micrometers could facilitate bone ingrowth and maintain the stability of the defect site.^268,269^ There are various methods for preparing pores with diameters of hundreds of micrometers, and the preparation method has a great influence on the porous structure and the final performance of bone biomaterials.^270,271^

The methods of preparing the porous structure of bioactive ceramics and biodegradable polymers mainly include gas foaming,^272,273^ space holding,^274^ freeze-drying,^275,276^ polymer sponge replication,^85^ thermally induced phase separation,^277–279^ solvent casting,^280^ and electrospinning.^281^ Tripathi *et al*^282^ prepared porous HA by the polymer sponge replication method. It had interconnected oval pores with diameters of 100–300 μm and a wall thickness of ~50 μm. Cell culture tests showed that osteosarcoma cells could adhere to and differentiate well on the porous HA and grow into the pores. Moreover, the cells exhibited better viability and differentiation on porous HA than dense HA due to the improved protein adsorption of the porous structure. Kaufmann *et al*^283^ investigated the effects of different porous structures of 45S5 on osteoblast proliferation and differentiation. The results showed that with a given porosity of 44%, pore diameter had no influence on the **in vitro** expression of osteoblasts, whereas with an average pore diameter of 92 μm, cell viability was influenced by porosity. Ma *et al*^284^ reported porous HA prepared by electrophoretic deposition. The pores were interconnected with a pore diameter ranging from several micrometers to several hundred micrometers. Cell culture results demonstrated that the bioactivity of porous HA was related to both the pore interconnectivity and diameter. Shin *et al* used electrospinning to prepare porous PCL and investigated its cytocompatibility by seeding it with mesenchymal stem cells (MSCs).^285^ After 1 week of culture, the cells had penetrated the pores, and a large amount of ECM had been generated. After 4 weeks of culture, the surface of the PCL was completely covered by cell layer. Oh *et al* prepared cylindrical porous PCL with a pore diameter increasing from 88 to 405 μm and a porosity varying from 80 to 94% along the axial direction.^286^ Subsequently, the effect of pore diameter on cell activity was assessed using chondrocytes, osteoblasts, and fibroblasts. A pore diameter of 380–405 μm allowed for better chondrocyte and osteoblast growth, while a pore diameter of 186–200 μm was better for fibroblast growth. Moreover, a pore diameter of 290–310 μm showed faster bone formation than other pore diameters. Although these methods can be used to prepare the porous structure of bone biomaterials, there are some remaining problems. For example, solvent casting has the disadvantages of monotonous pore diameter and solvent residue;^287^ thermally induced phase separation allows for little control over the pore diameter and interconnectivity;^37^ and gas foaming does not allow for control over the pore diameter, and it is difficult to prepare a pore diameter >200 μm by this method.^37^ Most importantly, these methods cannot facilitate the preparation of a material that mimics the complex internal structure of natural bone, limiting their application in bone repair.

Rapid prototyping is an advanced manufacturing technology, and its use is considered a milestone in the manufacturing field.^288^ Based on the discrete-stacking principle, rapid prototyping can achieve the preparation of arbitrary shapes and structures, and has thus received increasing attention in the preparation of porous bone biomaterials.^265,287,289,290^ Current rapid prototyping technologies mainly include selective laser sintering (SLS),^291,292^ stereolithography (SLA),^293,294^ and fused deposition modeling (FDM).^295^ Duan *et al*^296^ fabricated ceramic/polymer composites using SLS and found that the composites had a controllable microstructure, fully interconnected porous structure, and high porosity. Kim *et al*^297^ reported that the osteogenic signal expression of BMSCs could be improved effectively by adjusting the pore diameter of the biomaterials prepared by SLA. Zein *et al*^298^ fabricated porous PCL by FDM and found that it had a honeycomb and highly interconnected porous structure, with a pore diameter of 160–770 μm and a porosity of 48%–77%. Bose *et al* also used FDM to fabricate TCP ceramics with a pore diameter of 300–480 μm and a pore volume of 29%–44%.^299^ Cell culture experiments indicated that the pore volume had a certain influence on cell growth and a significant influence on the mechanical properties of TCP. Salmoria *et al*^300^ fabricated porous PCL by SLS and revealed that the pore diameter and porosity could be controlled by adjusting the laser parameters.

**Figure 5:**
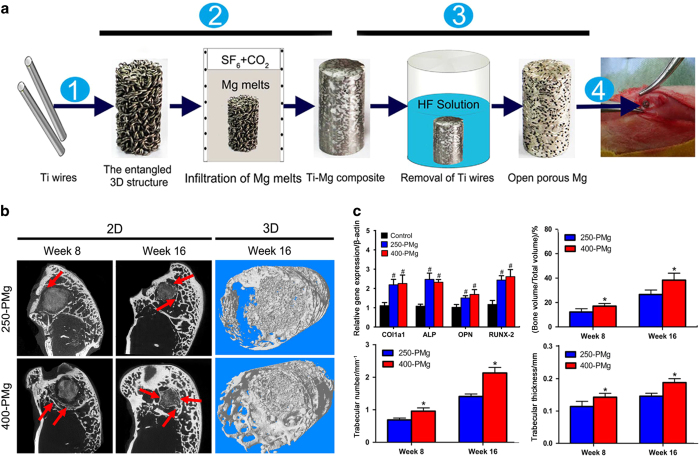
(**a**) The process of preparing porous Mg and an **in vivo** animal model. Step 1: an entangled 3D structure was prepared with Ti wires. Step 2: a Ti-Mg composite was prepared by the infiltration of Mg melts. Step 3: Ti wires were removed by hydrofluoric acid solution, yielding porous Mg. Step 4: the porous Mg was implanted into the lateral epicondyle of rabbits. (**b**) Characterization of the porous Mg and newly formed bone by micro-CT in 2D (red arrows) and 3D (white in color) reconstructions, showing a faster degradation and more bone regeneration for 400-PMg than 250-PMg at both time points. Here, 250-PMg and 400-PMg refer to porous Mg with a pore diameter of 250 and 400 μm, respectively. (**c**) Osteogenic differentiation and quantitative analysis of bone volume fraction and trabecular number and thickness, indicating a more densely packed bone structure for 400-PMg than 250-PMg.^315^

As for Mg and its alloys, the porous structure is mainly prepared by space holding,^301,302^ mechanical drilling,^303–305^ laser drilling^306,307^, and selective laser melting (SLM).^308^ Among these methods, mechanical drilling and laser drilling can only prepare pores with a monotonous shape and structure, as well as a narrow pore diameter distribution and low porosity.^309^ Space holding is one of the most commonly used methods but is also limited because of the residue of space holding materials and the lack of control over the porous structure.^310^ Currently used space holding materials include sodium chloride,^311^ ammonium bicarbonate,^244,312^ urea,^313^ and Ti wires.^314^ SLM possesses unique advantages in the preparation of complex internal structures but has high processing and equipment requirements. Therefore, its use is still in the experimental stage. Cheng *et al*^315^ reported that bone biomaterials with a pore diameter >100 μm were beneficial for osteogenesis and vascularization and that the porosity should be >50%. They prepared porous Mg with a porosity of 55% using Ti wires with diameters of 250 and 400 μm as a space holding material (Figure 5a). **In vivo** experiments indicated that at the same porosity, Mg with a larger pore diameter could accelerate early vascularization and gene expression, thereby promoting new bone growth (Figure 5b and c). Kirkland *et al*^316^ developed a technique combining rapid prototyping and gravity casting to prepare topographically ordered porous Mg, where a porous sodium chloride (NaCl) mold was first created, and then Mg was cast into the mold. After removing the NaCl, porous Mg with a porosity of 41% and a pore diameter of 1 mm was obtained. It can be concluded that the porous Mg and alloys developed so far have a porous diameter ranging from 100 μm to 2 mm and a porosity ranging from 20% to 80%. Moreover, the pore diameter and porosity have significant influences on the mechanical properties and degradation rate. For example, dynamic immersion experiments indicated that the degradation rate of porous Mg increased rapidly with porosity, and in combination with the porous structure, this markedly deteriorated the mechanical stability of Mg, which decreased by 89% after only 3 days of immersion.^317^ Zhang *et al*^318^ also found that as the porosity increased from 33 to 54%, the compressive strength of porous Mg decreased from 30.3 to 11.1 MPa, which was far below the requirement for load-bearing bone.

### Pore diameters of tens of micrometers

**Figure 6:**
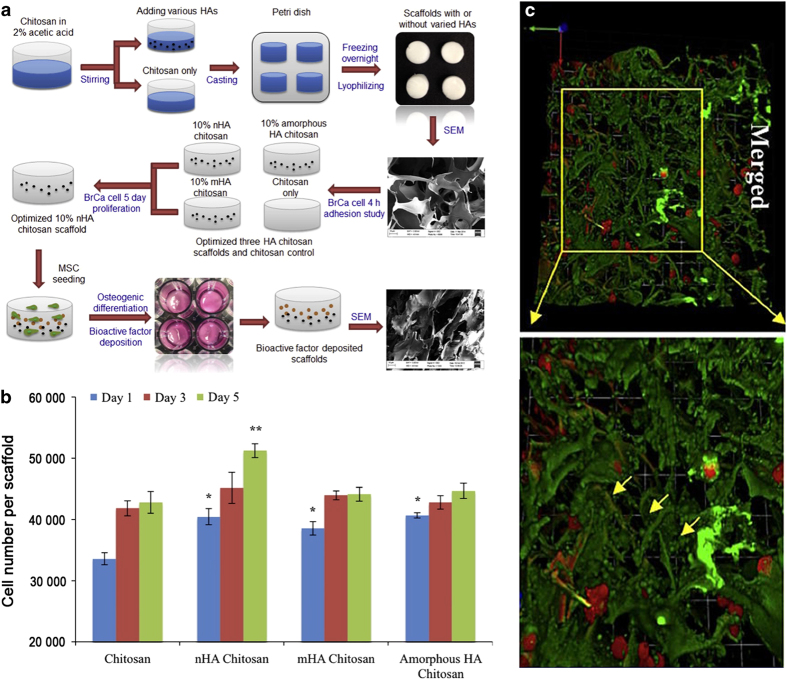
(**a**) Schematic illustration of MSC-modified chitosan bone model with different contents of microcrystalline HA (mHA), nanocrystalline HA (nHA), and amorphous HA fabricated by freeze-drying. SEM images showed a macroporous topography akin to that of cancellous bone with pore diameters of tens of micrometers. (**b**) Improved cell proliferation in the 10% nHA/chitosan bone model after 1, 3, and 5 days. *P<0.01 compared with the chitosan controls at day 1, and *P<0.01 compared with all other scaffolds at day 5. (**c**) Confocal microscopy images of cell distribution (red color) in the 10% nHA/chitosan bone model (green color) after 24 h. High-magnification image showing the deposited ECM components.^322^

Currently, the methods for preparing pore diameters of tens of micrometers mainly include rapid prototyping, freeze-drying, and sol-gel fabrication. Marques *et al*^319^ prepared porous glass by a sol-gel foaming process. The pores were spherical with numerous circular interconnections between neighboring pores. Cox prepared porous HA by 3D printing.^320^ The surface showed micropores (10–60 μm) and numerous topographical irregularities, which were considered beneficial for osteoconduction and osteointegration **in vivo**. Liu *et al*^321^ prepared porous HA/silica sol/sodium tripolyphosphate composites by rapid prototyping. It was found that the surface pores (5–25 μm) were suitable for the adhesion and growth of osteoblast-like cells. Zhu *et al*^322^ reported a 3D porous chitosan/HA composite with an average pore diameter of 20 μm fabricated by freeze-drying (Figure 6a). It was revealed that the tunable chitosan/HA ratio of the composite could provide a biologically relevant microenvironment, thereby increasing the cell–cell and cell–matrix interactions, as found in natural bone (Figure 6b and c). Jelen *et al*^323^ developed discrete functionally graded biomaterials by a cross-linking process to mimic the graded structure of natural bone. The pore diameter ranged from several to tens of micrometers. Mechanical characterization of the graded biomaterials showed a marked anisotropy, as in cancellous bone. Du *et al*^324^ prepared a series of 3D HA/PCL composites by SLS. The composites had uniform multi-scale porosity with pore diameters of 30–100 μm, as well as moderate mechanical properties and good biocompatibility. Nedjari *et al*^325^ prepared nanofibrous biomaterials with a gradient pore diameter ranging from 80 to 360 μm, depending on the honeycomb pattern size of the collector, which was beneficial for the regeneration of various bone tissues. A 3D macroporous nanowire nanoelectronic scaffold with a pore diameter of ~20 μm was also prepared to mimic the structure of natural bone.^326^ The results showed that the scaffold exhibited robust electronic properties and could be used alone or combined with other bone biomaterials as a biocompatible extracellular platform for the 3D culture of neurons, cardiomyocytes, and smooth muscle cells.

### Pore diameters of submicro- and nanometers

**Figure 7:**
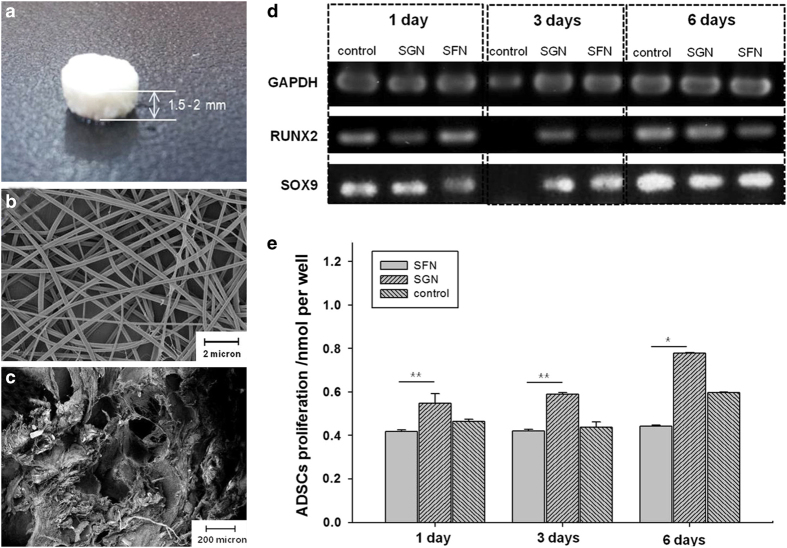
(**a**) Image of the SGN 3D biomaterials. SEM images of (**b**) the SGN used and (**c**) the porous structure of the SGN 3D biomaterials. (**d**) Gene expression analysis of an osteoblast gene marker (runt-related gene 2 (RUNX2)) and a fibrocartilage gene marker (SOX9) after 1, 3, and 6 days of cell culture under control conditions (ADSCs seeded in culture wells without a scaffold) or in the presence of the SGN 3D biomaterials. (**e**) Water-soluble tetrazolium salt proliferation assay of ADSCs under control conditions or in the presence of the SGN 3D biomaterials for 1, 3, and 6 days (*P<0.05, **P<0.01).^329^

Despite the considerable efforts that have been made to develop macro/microporous bone biomaterials and study their interactions with cells, porous biomaterials should also have nanofeatures to better match the nano-architecture of the ECM.^327^ An attempt was made to develop porous nanofibrous PLLA with a pore diameter down to the nanometer scale by the liquid–liquid phase separation method.^328^ Cell culture tests showed that the nanostructured PLLA acted as a positive cue to support stem cell differentiation and tissue growth, suggesting that the nanostructured porous PLLA has potential as a cell carrier in bone repair. Another study constructed a silkworm gland nanofibrous (SGN) biomaterial by electrospinning (Figure 7a).^329^ SEM images revealed that the biomaterial had a homogenous pore distribution, with nano/micropores of (219±39) nm and (201±22) μm in diameter, respectively (Figure 7b and c). Moreover, the biomaterial also showed well-defined interconnected pores, with a porosity of 85.3%±2.5%. In subsequent biological tests, the SGN 3D biomaterials efficiently attenuated the oxidative stress-induced cell damage and promoted the proliferation of adipose tissue-derived stem cells (ADSCs) while maintaining their cell lineage phenotypes (Figure 7d and e). A micro/nanohybrid surface structure was also fabricated on porous HA via hydrothermal treatment.^330^ The results showed that the micro/nanotopography of the surfaces significantly enhanced the cell adhesion and viability, alkaline phosphatase (ALP) activity, and mRNA expression levels of both the osteogenic markers and angiogenic factors of ADSCs. These findings suggested that the hierarchical micro/nanohybrid surface topography could act as a stem cell carrier. In addition, photolithography has also been applied to fabricate biomaterials with multiple types of micro/nanotopography for bone repair.^331^ The results demonstrated that pores with a suitable micro/nanotopography could guide and promote the responses of endothelial cells. Nanoscale pores were also introduced onto the surface of porous PLGA by chemical etching.^332^ The results showed that the nanoscale surface features promoted cell adhesion and growth, as well as elastin and collagen production. Recently, Kang *et al*^333^ designed an electrospun polyethylene oxide/PCL biomaterial with mesoporous bioactive nanocarriers to sequentially deliver two growth factors. The prepared biomaterial showed enlarged mesopores of ~7 nm, with a large surface area and pore volume. **In vitro** and **in vivo** experiments revealed that the mesoporous biomaterial acted as an efficient carrier for the long-term delivery of growth factors, thereby significantly promoting cell proliferation and bone formation. These studies revealed that the integration of nanofeatures into bone biomaterials could provide better control over cell functions via cell–nanofeature interactions.

## Interactions between bone biomaterials and stem cells

In bone repair, bone biomaterials provide a platform for supporting stem cell adhesion and growth. It has been found that the chemical composition, surface properties, and topographical structure of bone biomaterials can directly affect the adhesion, proliferation, and differentiation of stem cells, as well as the formation of neovascularization networks. Stem cells are responsible for the osteogenic commitment and maturation of osteoblasts by secreting matrix components and promoting calcification, that is, the formation of new bone. In the above sections, we have introduced the research progress of bone biomaterials, and we will now discuss the seed stem cells and signaling pathways involved in the adhesion and osteogenic differentiation of stem cells mediated by biomaterials for bone repair.

### Sources of stem cells

In recent years, stem cell-based regenerative medicine has unfolded as a new field of biomedicine. Because of their good proliferation ability and pluripotency, stem cells are increasingly used to accelerate wound healing and recover the function of impaired tissues and organs through stem cell transplantation and differentiation.

#### Embryonic stem cells (ESCs)

ESCs derived from the inner cell mass of blastocyst-stage embryos are very proliferative in their undifferentiated state. ESCs are the stem cells with the most potential.^334^ They have pluripotent characteristics, including the ability to undergo osteogenic differentiation.^335–339^ Tang *et al* first seeded ESCs into alginate microbeads in macroporous calcium phosphate cement and implanted the constructs into nude mice. Good cell viability, osteogenic differentiation, and mineral synthesis were demonstrated.^340^ Rutledge *et al* reported that porous PLGA with and without an ECM coating increased ESC proliferation and osteocalcin expression.^341^ However, the application of ESCs faces three major issues: ethical constraints, safety issues, and the tight control of **in vitro** conditions to regulate osteogenic differentiation.^342^

#### MSCs

MSCs are important members of the stem cell family and the most established and investigated stem cell type. MSCs are isolated from the early-stage mesoderm and ectoderm, and they are pluripotent. They were originally found in the bone marrow.^343^ Later, they were found to be abundant in the bone, adipose tissue, and synovial membrane, among other tissues. They have excellent potential in bone repair because of the following characteristics: (1) They have excellent proliferation and differentiation potential both **in vivo** and **in vitro**, and they can differentiate into any cell type, including osteoblasts and muscle cells. (2) They have a regulatory immune function; they can inhibit the proliferation and immune activity of T cells through the interactions between cells and cytokines. (3) They have the advantages of being conveniently sourced and easily separated, cultured, amplified, and purified. Additionally, MSCs maintain the characteristics of stem cells after multiple passages without immunological rejection. (4) They have low antigenicity and light allograft rejection, and the matching requirement is not strict. Thus far, several types of MSCs have been investigated for osteogenic differentiation.

Among various MSCs, BMSCs were first found and isolated and are currently the most commonly used MSCs in experimental and clinical trials. Because they are easily sourced, isolated and cultured and exhibit a strong potential for differentiation and autologous transplantation, they are considered optimal for use in clinical stem cell treatments.^344,345^ Recently, Berglund *et al*^346^ found that an Mg–Ca–Sr alloy possessed advantageous characteristics, including good mechanical strength and degradation behavior, and displayed potential osteogenic properties, which were characterized as ALP expression and RUNX2 activation. Yu *et al*^347^ found that mesoporous HA microspheres not only enhanced the expression of osteogenic markers in BMSCs but also promoted the migration and tube formation of EA.hy926 cells. In addition, PCL and PLA nanofibrous scaffolds were reported to be safe and non-toxic and could support the adhesion and proliferation of BMSCs.^348^

Umbilical cord (UC)-MSCs recently have gained attention because of their strong proliferation ability and multilineage differentiation potential.^349,350^ They have a better proliferation ability and lower immunogenicity and are more conveniently available than BMSCs. Furthermore, there is no ethical controversy surrounding their use. They have broad clinical applications in the regeneration of bone, cartilage, and muscle, among other tissues. Nano-HA/chitosan/PLGA scaffolds induced human UC-MSCs to differentiate into osteoblasts for the repair of calvarial defects in nude mice.^351–353^

Dental pulp stem cells (DPSCs) were originally isolated from human dental pulp by Gronthos in 2000.^354^ He found that DPSCs had similar characteristics, such as osteogenic ability and immunophenotype, to BMSCs, but a better proliferation ability. DPSCs have the capacity for self-renewal and the potential for multilineage differentiation into a variety of cell types. They can differentiate into cells found in bone, cartilage, vascular endothelium, and others through induction using different cytokines.^355^ They can also be used in the treatment of a variety of diseases by regulating immunity and anti-aging mechanisms. The attractive advantages of DPSCs can be summarized as follows: (1) there is a rich source of DPSCs in the naturally shed teeth of children 6–11 years of age, as well as in the wisdom teeth of adults, which are often removed and discarded as wastes. (2) Minor side effects: DPSCs are a type of MSCs with low immunogenicity, which would not be rejected when transplanted. (3) No ethical controversy: As DPSCs are derived from the naturally shed teeth of children and the wisdom teeth of adults, there would be no side effects for the donor. The differentiation and regeneration ability of DPSCs are impressive, suggesting the potential use of DPSCs for bone repair and regeneration.^356,357^ Therefore, DPSCs have gained increasing attention due to their favorable biological characteristics.

DPSCs were seeded on 3D TCP/poly(l-lactic acid/caprolactone) biomaterials and cultured in osteogenic medium containing vitamin D3 for 14 days. The results showed that DPSCs can be induced to differentiate into osteoblasts, indicating good osteogenic potential.^358^ Tricalcium silicate cement increased the expression of mineralization-associated genes (collagen type I alpha 1 (COL1A1), ALP, dentin sialophosphoprotein (DSPP), and RUNX2).^359^ Jensen *et al*^360^ reported that the functionalization of porous PCL with hyaluronic acid and TCP facilitated the migration and osteogenic differentiation of human DPSCs **in vitro**.

#### Induced pluripotent stem cells

Since Takahashi obtained induced pluripotent stem cells (iPSCs) by the transduction of transcription factors,^361^ iPSCs have shown wide prospects in bone repair. iPSCs exhibit potentially unlimited proliferation and pluripotent differentiation with no ethical controversies; thus, they are now considered an attractive option for osteogenic differentiation and bone regeneration. Recent discoveries have demonstrated that iPSCs could differentiate into osteoblasts.^362^ The differentiation of ESCs or iPSCs into bone cells has been adapted from the protocols for the osteogenic differentiation of MSCs. The basic components of the commonly used osteogenic medium are fetal bovine serum, ascorbic acid, β-glycerophosphate, and Dex. Additional enhancing supplements include certain bone morphogenetic proteins (BMPs) or the calcium-regulating hormone vitamin D3. Recently published data offers some indications of bone-like and mineralized tissue formation by iPSCs **in vivo**,^363,364^ suggesting that iPSCs have the potential to be applied in bone repair.

#### Osteoblasts derived through transdifferentiation

Transdifferentiation occurs when one type of differentiated cell is transformed into another type of differentiated cell.^365^ This is a technique that has been developed in recent years, and osteoblasts obtained by transdifferentiation through transcription factors, cytokines, small molecules, microRNA, and epigenetic regulation are emerging. Tansriratanawong *et al*^366^ reported that dedifferentiated fat cells (DFATs) revealed osteogenic differentiation when co-cultured with periodontal ligament stem cells. They found that DFATs attenuated proliferation while enhancing osteogenic gene expression (RUNX2); however, their adipogenic characteristics diminished. DFATs and the co-culture system might be a novel cell-based therapeutic method for promoting osteogenic differentiation in periodontal regeneration. Cho *et al* reported that the epigenetic modification of Wnt3a by BMP-2 revealed a new mechanism in the morphogen-mediated control of osteogenesis. In their study, the CpG-demethylating agent 5-aza-2-deoxycytidine or the histone deacetylase inhibitor trichostatin A was used to decrease the methylation and increase the acetylation of histone H3 lysine 9 specifically in non-osteogenic cells, which contributed to the direct transdifferentiation of pre-adipocyte fibroblasts into osteoblasts.^367^ Though at the primary stage, this strategy would be promising for application in bone repair.

### The effect of biomaterials on stem cells

**Figure 8:**
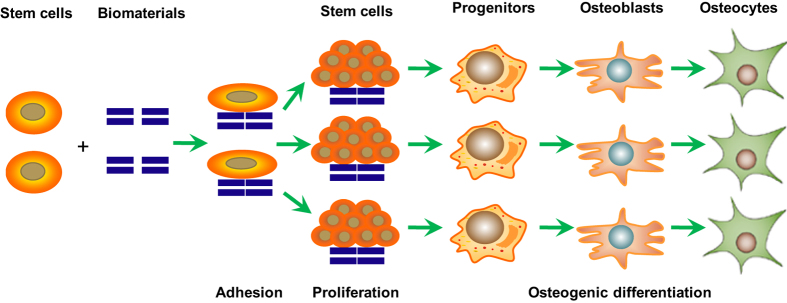
The interactions between bone biomaterials and MSCs.

While many sources can be selected, the potential of different cells differs from one another. To induce the osteogenic differentiation of stem cells, a befitting; microenvironment is required. Ideal biomaterials for bone repair should have the following characteristics: (1) osteoconductivity, which can provide a place for blood vessel formation and bone ingrowth with a certain mechanical strength; (2) osteoinductivity, which can induce the expression of osteogenic proteins and stimulate surrounding stem cells to differentiate into chondrocytes or osteoblasts, followed by mineralization and calcification until new bone formation is achieved; and (3) osteogenesis, which can induce the differentiation of progenitor cells, osteoblasts, and bone progenitors into osteoblasts or their maturation. The interactions between bone biomaterials and stem cells include three steps: adhesion, proliferation, and differentiation (Figure 8).

#### The adhesion and proliferation of stem cells

Cells, including stem cells, can sense multiple extracellular signals from their microenvironment and simultaneously convert them into coherent environmental signals to regulate cell behavior. Nonspecific adhesion generally occurs through van der Waals, ionic, and electrostatic forces. In comparison, specific adhesion is mediated by the ECM, including collagen I, fibronectin, peptides, growth factors, glucosamine, and other active molecules, and is induced by activating receptors on the surface of the cell membrane and transducing the chemical signal into the cell, thereby modulating a series of biological cellular activities.^368,369^ Bone biomaterials can affect the secretion of the ECM and act as an ECM to interact with stem cells.

**Figure 9:**
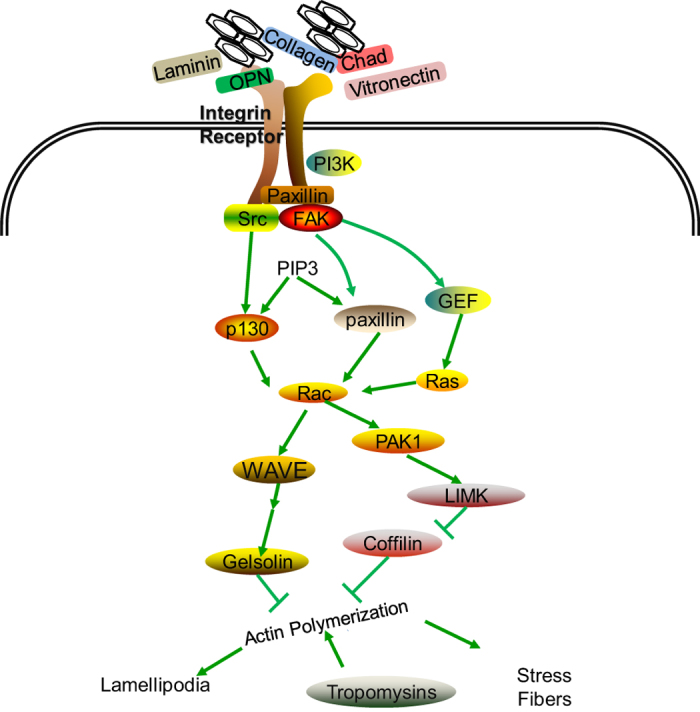
The signaling pathway in the adhesion of stem cells to bone biomaterials.

The cell adhesion process consists of a series of cascaded reactions, which can be divided into four steps: cell adhesion, cell spreading, cytoskeletal organization, and the formation of focal adhesions (Figure 9).^370^ Initial adhesion plays a critical role in cell differentiation and long-term stability.^371^ The initial adhesion between cells and biomaterials is mediated by ionic bonding or van der Waals forces, and the interaction is fast and transient. The adhesion promotes the interaction between the ECM and the integrin or transmembrane receptor, resulting in the formation of focal adhesion plaques. The cytoplasmic domain of the integrin receptor links to some adaptor proteins (for example, talin, vinculin, tensin, and paxillin), which further link to actin filaments. The ECM–integrin–cytoskeleton axis mediates the adhesion process, which not only regulates cell-biomaterial adhesion but is also responsible for the signal transduction from outside of the cell to within the cell membrane (Figure 9). Integrins link the ECM with the cytoskeleton by focal adhesion components and activate focal adhesion kinase (FAK) and Src kinase. FAK phosphorylates two other groups, paxillin and Crk-associated substrate (p130cas), which enable the bonding of the signal adaptor protein with the focal adhesion.^372,373^ This process is accompanied by actin assembly and FAK dynamic changes, which are involved in cell adhesion, spreading, invasion, proliferation, and apoptosis. Cell adhesion can also activate other intracellular signaling pathways, such as the mitogen-activated protein kinase (MAPK) signaling pathway.^374–376^

Cell-ECM adhesion is mediated by the binding of ligands (including elastic fibers, collagen family members, proteoglycans, glycosaminoglycans, and adhesive glycoproteins) to the integrin receptors.^370^ Apart from the abovementioned ligands, the ECM also contains organic materials with excellent mechanical and biological properties. Currently, bioactive ceramics, as well as biodegradable polymers and biodegradable metals, are considered promising biomaterials in bone repair. As organic materials, biodegradable polymers can act as an ECM for cell attachment, and adhesion is promoted by combining the polymer with ligands.^377,378^ Natural polymers, such as albumin, alginate, amylose/amylopectin, chitosan, collagen, elastin, fibrin, fibronectin, hyaluronic acid, keratin, and silk, are applicable and can promote cell adhesion.^379^ For better cell adhesion, polymers are often coated with fibronectin or laminin, while bioactive ceramics are often coated with collagen I/III or fibronectin.^380^ It remains a technological challenge to prepare topographical gradients of inorganic biomaterials because of their inherent material properties.^381,382^ The use of biodegradable metals in direct contact with MSCs has not yet been reported. However, their surfaces were coated with an ECM-like serum from the medium and hence have shown relatively good compatibility.^383–385^ At the same time, the identification of some novel ligands, such as FHOD1 and CD82, has also inspired exploration of the mechanism of cell–matrix adhesion and cell migration in 3D microenvironments.^386–388^

#### The osteogenic differentiation of stem cells

A 3D implant and seeded cells form a local microenvironment, which consists of various chemicals, mechanical stresses, and growth factors. These components act as active molecules and activate the adhesion/proliferation/differentiation signaling pathways for the adsorption of stem cells onto biomaterials. Shih *et al*^389^ found that calcium phosphate induced the differentiation of stem cells into osteoblasts (bone-building cells) by the following mechanism: phosphate ions dissolved from these biomaterials were absorbed by stem cells to form important metabolic molecules (for example, ATP), and then the ATP metabolites (adenosine) sent signals to stem cells and induced them to differentiate into osteoblasts. They also reported that the small-molecule adenosine can induce stem cells to differentiate into osteoblasts.^390^ Porous titanium dioxide (TiO_2_) coated with alginate hydrogel containing various concentrations of simvastatin induced the osteogenic differentiation of human adipose tissue-derived (hAD)-MSCs. COL1A1, ALP, osteopontin (OPN), osteocalcin (OCN), and vascular endothelial growth factor (VEGF) A were enhanced in hAD-MSCs.^391^

Ren *et al*^392^ described a nanoparticulate mineralized collagen glycosaminoglycan implant, which induced the healing of critical-sized rabbit cranial defects without the addition of expanded stem cells or exogenous growth factors. This strategy may provide novel growth factor-free and *ex vivo* progenitor cell culture-free implants for bone repair. Luo *et al* also reported graphene oxide (GO)-doped PLGA nanofibers prepared by electrospinning for enhancing the osteogenic differentiation of MSCs. They demonstrated that GO not only enhanced the hydrophilic performance and protein- and inducer-adsorption abilities of the nanofibers but also accelerated the adhesion, proliferation, and osteogenic differentiation of human MSCs (hMSCs).^393^ Our research group and Li *et al* almost simultaneously reported that boron nitride nanotubes could promote the differentiation of MSCs into osteoblasts, which was confirmed in animal models.^394,395^

Although some biomaterials (mainly inorganic materials) have been reported to be capable of inducing the osteogenic differentiation of pluripotent stem cells independent of cytokines or growth factors, most of the relevant molecular mechanisms are still not well defined.

### The mechanisms by which biomaterials initiate or enhance the osteogenic differentiation of stem cells

The biphasic calcium phosphate ceramic-mediated secretion of signaling molecules, inflammatory factors (interleukin 1, interleukin 6, and monocyte chemoattractant protein 1), and growth factors (VEGF, platelet-derived growth factor, and epidermal growth factor) by macrophages were upregulated and promoted cell migration and the gene expression of osteogenic markers [ALP, collagen type I, osterix (OSX), bone sialoprotein, and OPN].^396^ Low-magnitude, high-frequency vibrations promoted the adhesion and osteogenic differentiation of BMSCs cultured on a HA-coated surface through Wnt/β-catenin signaling.^397^ Hao *et al*^398^ found that compared with –OEG and –CH_3_ groups, –PO_3_H_2_, –OH, –NH_2_, and –COOH on the self-assembled alkanethiol monolayers on gold promoted not only cell adhesion, proliferation, and osteogenic differentiation but also the expression of α_v_ and β_1_ integrins. Graphene/CNT hybrids promoted the osteogenic differentiation of MSCs by activating the p38 signaling pathway.^399^ Mg ions resulting from the degradation of bone biomaterials can promote the release of neurotransmitters [for example, calcitonin gene-related peptide (CGRP)] in the periosteal part of the sensory nerve terminal; the increased CGRP level further promotes the periosteal osteogenic differentiation of stem cells and the final formation of large amounts of new bone in periosteal regions.^400^ Sr ions can enhance the osteogenic differentiation of MSCs and **in vivo** bone formation by activating the BMP signaling pathways.^401,402^

**Figure 10:**
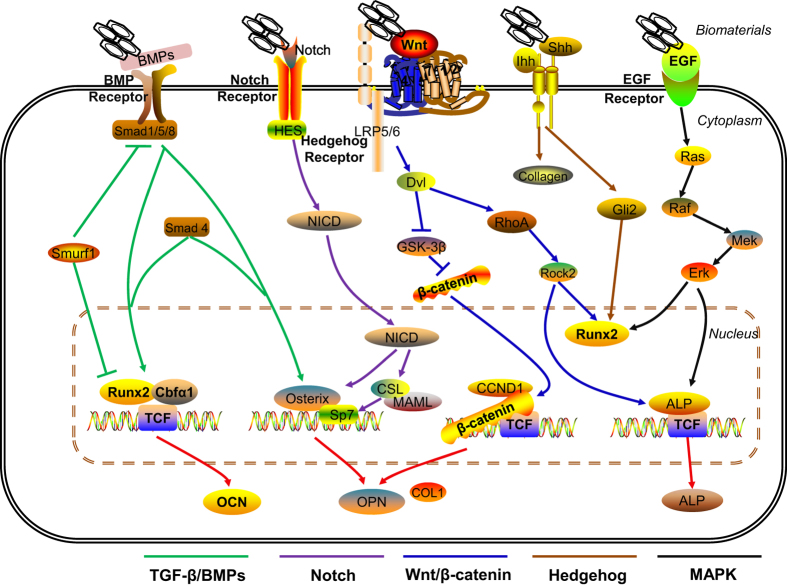
The main signaling pathways in the osteogenic differentiation of MSCs induced by biomaterials.

The ECM is an active and complex microenvironment with outstanding biomechanical, biophysical, and biochemical characteristics, which can indirectly or directly influence cell adhesion, migration, proliferation, and differentiation, as well as tissue and organ regeneration. Biomaterials mimicking the tissue-specific physicochemical properties of the ECM are being developed at a rapid pace to regulate stem cell fate. The activation of respective receptors responding to different ligands leads to the activation of different signaling pathways involved in the biomaterial-induced osteogenic differentiation of stem cells, as illustrated in Figure 10.

#### Transforming growth factor *β*/BMP pathway

BMPs have a high affinity for the type I receptor BMPR1A and a low affinity for the type II receptor BMPR2. Transforming growth factor β (TGF-β) activates TGF-β signaling through binding to TGFBR2 and TGFBR1. The activation of these two receptor classes further promotes the phosphorylation of Smad proteins. TGF-β signaling has a complex effect on bone formation. It can induce synthesis of the ECM and promote the differentiation of osteoblasts; additionally, it is associated with the chemotaxis of osteoblast-like cells to the ECM and the recognition of target cells. BMPs were first isolated from a mineralized bone matrix extract, and they can induce the differentiation of the undifferentiated MSCs into bone cells, followed by collagen synthesis and calcified bone tissue formation. BMP-2, BMP-4, BMP-5, and BMP-7 can effectively promote the differentiation of BMSCs into osteoblasts and induce bone formation; among them, BMP-2 exhibits the strongest activity. There are BMP receptors on the cell membrane. These receptors are activated by BMP binding or mechanical stimulation and then act on the downstream Smad protein in the cytoplasm. Smad protein is an intracellular signal transduction protein that was recently discovered. Among the family members, Smad1 and Smad5 are associated with osteoblast differentiation. Smad1, Smad5, and Smad8 terminal serine residues are phosphorylated by BMP receptors, and then heterotrimers or heterodimers formed by 2 or 1 R-Smad and Smad4 enter the nucleus, activating osteoblast-specific transcription factors (RUNX2 and OSX) and corresponding downstream targets.^403,404^ In fact, there are some applications of BMP-2 as an important cofactor to support bone regeneration on various scaffolds. BMP-2-loaded biphasic calcium phosphate significantly promoted the ratio of bone volume/tissue volume **in vivo** compared with that without BMP-2.^405^

#### Wnt/*β*-catenin signaling pathway

The Wnt/β-catenin signaling pathway is associated with organ development, especially osteogenic differentiation. Wnt protein comprises a series of highly conserved secretory glycoproteins. A total of 19 members have been found, and the different members have unique secretory patterns and play different roles in embryonic development. Wnt protein is composed of 350–380 amino acids and can be divided into classical Wnt proteins (Wnt1, Wnt2, Wnt3, and Wnt3a) and non-classical Wnt proteins (Wnt4, Wnt5a, Wnt5b, Wnt6, Wnt7a, and Wntl1, among others). The classical Wnt proteins interact with the Frizzled/LRP (Fzd/LRP) receptor and activate the Wnt/β-catenin classical pathway, while non-classical Wnt proteins bind to the Fzd receptor, activating heterotrimeric G protein and regulating the intracellular level of Ca ions. The combination of Wnt protein with the Fzd/LRP receptor activates the second messenger, causing the accumulation of cytoplasmic β-catenin. The accumulated β-catenin subsequently enters the nucleus and interacts with T cell transcription factor and lymphoid enhancer factor, which bind to the promoters of RUNX2 and OSX and activate their transcription.^406^

#### MAPK signaling pathway

Among the MAPK family members, extracellular signal-regulated kinase (ERK), p38, and c-Jun N-terminal kinase (JNK) are involved in the signal transduction of hMSC osteogenic differentiation. The ERK pathway can be activated throughout the development of stem cells and is closely related to cell proliferation and differentiation, while the p38 and JNK signaling pathways may play a role in the late stages of cell differentiation or apoptosis. The current mechanism of the ERK pathway is well defined. When in contact with osteoblast precursors, the ECM can bind with the integrin receptor of the cell surface and induce receptor dimerization, autophosphorylation, and FAK activation. As a result, FAK activates Ras, which further promotes transcription factors to initiate the expression of specific genes through conservative enzymatic cascades. It is known that ERK5 and ERK1/2 can activate the expression of the immediate early genes c-Fos and c-Jun.^407^ In addition, when hMSCs are added to the culture medium, the activity of ERK is activated after 7–11 days, and the differentiation of hMSCs is promoted by c-Jun. JNK is activated after 13–17 days, and ECM synthesis and calcium deposition increases. Other non-collagenous proteins, such as laminin 5 and matrix protein 3, can induce the expression of Cbfa-1 and ALP through the ERK1/2 signaling pathway and can enhance the matrix mineralization and osteogenic differentiation of hMSCs.^408^ Together, these results suggest that the MAPK signaling pathway plays an important role in the osteogenic differentiation of hMSCs.

#### Hedgehog signaling pathway

The activation of the Hedgehog signaling pathway can promote the osteogenic differentiation of BMSCs through the key downstream molecules Smoothened (Smo) and Gli1, and then activate mTORC2-Akt signaling by IGF. Transport protein 80, located in the flagellar structure, activates canonical Hh-Smo-Ptch1-Gli signaling and inhibits non-classical Hh-G*α*i-RhoA signaling, and then promotes differentiation into osteoblasts. The Hedgehog signaling pathway can promote bone differentiation and matrix mineralization in combination with the BMP and Wnt signaling pathways, and an **in vivo** study using a bone transplantation model confirmed the effect of these signaling pathways on bone formation and bone defect healing.^409–411^

#### Notch signaling pathway

**In vitro** studies have confirmed that the activation of the Notch signaling pathway can inhibit the differentiation and promote the proliferation of osteoblasts. The persistent activation of Notch inhibits the differentiation of MSCs into osteoblasts, resulting in decreased bone mass. Kohn *et al*^412^ showed that Notch2 played a leading role in the inhibition of bone formation through activated Notch protein, the Notch intracellular domain (NICD), and RBPjkappa. Hilton *et al* showed that Hes and Hey proteins inhibited osteoblast differentiation and regulated the activity of OCN and OPN gene promoters through interacting with RUNX2. Some studies showed that overexpression of NICD at the cellular level stimulated the proliferation of osteoblasts in the early stages but prevented them from growing into mature osteoblasts.^413^ The Notch signaling pathway can also enhance the proliferation of osteoblasts through the upregulation of cyclin D and cyclin E.^414^

We introduced several signaling pathways that induce the osteogenic differentiation of MSCs by responding to different stimuli, including growth factors, cytokines, physical stresses, topographical features, and chemical factors. In fact, activation of any of the above signaling pathways will lead to osteogenic differentiation, and the combination of several pathways will promote and accelerate the process.

### The progress of vasculogenesis promoted by bone biomaterials

It is also important to promote vasculogenesis, especially in large segmental defects. Some of the biomaterials used for bone tissue engineering can stimulate the formation of new vessels. Although it has been widely investigated, how biomaterials affect the formation of new vessels is still not well understood. The several examples here provide some information about the advances in this field in recent years. Low concentrations of HA not only promoted bone regeneration but also positively affected the formation of an endothelial network in collagen/fibrin hydrogels **in vitro**. However, the responses were not obvious **in vivo**.^415^ β-TCP and calcium-deficient HA ceramics shared similar properties to promote the proliferation and differentiation of endothelial cells.^416^ Sr and Si ion-containing bioceramics exerted synergistic effects on osteogenesis, osteoclastogenesis, and angiogenesis due to the effects of Si ions on enhancing osteogenesis and the effects of Sr ions on enhancing angiogenesis **in vitro** and **in vivo**. Sr and Co ions improved both the osteogenesis and angiogenesis of human osteosarcoma cells and human umbilical vein endothelial cells co-cultured with bioactive glasses.^417^ Recently, a study demonstrated that Cu ion-containing bioactive glass significantly promoted the expression of the HIF-1a gene, which upregulated the expression of osteogenesis-related genes (including S100A10, BMP-2, and OCN) and angiogenesis-related genes (such as VEGF).^418,419^ Some advanced materials, such as graphene, were reported to promote the secretion of angiogenesis-related proteins, such as von Willebrand factor and angiotensin 1, as well as angiogenesis.^420^ More attention has been focused on the combination of several materials with different properties.^421–423^

## Summary and perspectives

The development of bone biomaterials to mimic the material properties and porous structure of natural bone is now under extensive investigation. These studies have focused on developing ideal biomaterials with a combination of satisfactory biological and mechanical properties, constructing a cell microenvironment with a multi-scale porous structure, and inducing the oriented differentiation of stem cells for artificial-to-biological transformation.

Regarding bone biomaterials, recent studies have focused on bioactive and/or biodegradable biomaterials, including bioactive ceramics, biodegradable polymers, and biodegradable metals. Bioactive ceramics have inorganic constituents similar to those of natural bone and possess excellent biocompatibility and bioactivity. However, their inherent brittleness and low toughness limit their application in bone repair. In comparison, biodegradable polymers possess a relatively high toughness and plasticity, and their performance can be modulated by molecular design and fabrication methods. However, there is a contradiction between the mechanical properties and degradation rate, while synthetic biodegradable polymers always have a poor cell affinity and much lower strength than natural bone. As biodegradable metals, biomedical Mg and its alloys are stronger than biodegradable polymers and tougher than bioactive ceramics. However, the rapid loss of mechanical integrity and the accumulation of corrosion products caused by rapid degradation have long been major problems for Mg and its alloys. Therefore, a single type of bone biomaterial can hardly meet the multiple requirements for application in bone repair. To meet the requirements of both the mechanical and biological properties, many researchers have tried to combine different types of bone biomaterials, hoping to combine the advantages of each biomaterial while overcoming their disadvantages.

As for the cell microenvironment, although many of its specific biological functions are still unclear, attempts to mimic the microenvironment by designing bone biomaterials with a porous structure have continued. Especially in recent years, the construction of a cell microenvironment has become a common focus of multiple disciplines, including engineering, medical, and materials sciences. On the one hand, there is a basic agreement about the functions of different pore diameters, and the relationship between the porous structure of bone biomaterials and cell behavior has been experimentally studied. However, the mechanisms of energy interactions and functions of the porous structure with respect to cells/tissues remain unclear. Related studies have focused on a single-scale porous structure, but the formation mechanism of a cell microenvironment with a multi-scale porous structure remains to be studied. On the other hand, in consideration of the different characteristics of bone biomaterials, various methods have been developed to prepare porous structures, including gas foaming and freeze-drying, among others. These methods can prepare materials with pore diameters ranging from hundreds of micrometers to nanometers but often lack control over the porous structure. The development of rapid prototyping resolves the above problems to a large extent, as it can be used to precisely control the pore diameter, porosity, and pore interconnectivity of bone biomaterials. However, it is limited by the available types and compositions of raw materials; additionally, it is difficult to prepare a submicro- and nanoscale porous structure. Therefore, the porous structures developed so far remain largely different from that of natural bone.

With the development of new stem cell-based techniques, there are many more choices of seed stem cells for bone repair, such as iPSCs and transdifferentiation-derived osteoblasts. However, the tumorigenic potential, limited sources, and low transdifferentiation efficiency of iPSCs are the main challenges surrounding their application. There are still many obstacles to overcome. In comparison, the potential of several MSCs, such as DPSCs and UC-MSCs, is encouraging, as they have rich sources, noninvasive harvesting techniques, and excellent self-renewal and proliferative capacities. Although the detailed molecular mechanisms regarding how bone biomaterials induce the osteogenic differentiation of stem cells are still largely unknown, some evidence has demonstrated that associated signaling pathways may be involved in the process. For example, the P ions dissolved from bone biomaterials can be absorbed by stem cells to form ATP metabolites, which send signals to stem cells and induce osteogenic differentiation. The release of Mg and Sr ions from bone biomaterials could enhance osteogenic differentiation through promoting the concentration of CGRP and activating the BMP signaling pathway, respectively.

Notwithstanding the intensive efforts and great achievements behind biomaterials and their interactions with stem cells, the bone biomaterials developed thus far still have some deficiencies, and there is plenty of room for improvement: (1) develop new preparation methods for bone biomaterials from the perspectives of histology, cytology, and molecular biology. According to different needs, filter the prepared bone biomaterials and seek the optimal combination; subsequently, develop composite bone biomaterials with satisfactory biological and mechanical properties. (2) Explore the mechanisms of energy interaction and functions of the porous structure with cells/tissues at the molecular level; reveal the formation mechanism of the microenvironment with the synergistic effect of the multi-scale porous structure; seek the optimal composition of a multi-scale porous structure, and thus provide theoretical guidance for the design and manufacture of the porous structure. (3) Develop cross-scale methods for preparing the multi-scale porous structure; the combination of several methods is a promising direction for preparing porous bone biomaterials. (4) Develop novel surface micro/nanostructures for bone biomaterials to improve their ability to provide biochemical signals for cells, thereby facilitating the oriented differentiation of stem cells and achieving artificial-to-biological transformation. (5) The molecular mechanism of stem cell lineage commitment by biomaterials has not been well defined. Thus, further efforts should be made to elucidate the differentiation mechanisms and seek novel biomaterials with better osteoinductivity. In summary, developing bone biomaterials with material compositions, geometric structures, and physiological functions similar to those of natural bone and investigating their interactions with stem cells for bone repair remain significant avenues for future research.
